# Subjective Well-Being in Cancer Patients: The Roles of Social Support, Purpose in Life, Resilience, and Informativeness

**DOI:** 10.3390/healthcare11243181

**Published:** 2023-12-16

**Authors:** Lovorka Brajković, Karla Milat-Panža, Vanja Kopilaš

**Affiliations:** Faculty of Croatian Studies, University of Zagreb, 10000 Zagreb, Croatia; lbrajkov1@hrstud.hr (L.B.);

**Keywords:** cancer, subjective well-being, social support, resilience, prosperity, informativeness

## Abstract

The aim of this research was to determine the relationship between subjective well-being (life satisfaction, positive and negative experiences, and prosperity) and various psychosocial factors (social support received from family members and partners, purpose in life, resilience and information) in cancer patients and to examine the possibility of predicting components of subjective well-being based on these mentioned factors in cancer patients. A total of 338 adult cancer patients from Croatia participated in the study (41.1% male and 58.9% female). To measure the constructs, the Diener Subjective Well-Being Scale, the Social Support Scale at work and in the family, the Purpose in Life Scale, the Short Resilience Scale, and the EORTC-QLQ information questionnaire were used. Results showed a high level of life satisfaction and prosperity, as well as more frequent positive compared to negative experiences. A medium to high level of social support received from family members and from the partner was determined. High levels of purpose in life and medium levels of resilience and information were found. A high correlation was found among the components of the construct of subjective well-being, and a low to medium correlation among the predictors. Positive associations were found between the criteria of life satisfaction and prosperity with psychosocial factors. Negative associations were established between the positive/negative experience variables and the factors. Furthermore, the purpose in life was determined as an important predictor of all three components of subjective well-being, social support (both sources) as important for predicting life satisfaction, resilience for experiencing positive and negative experiences, and social family support for predicting the prosperity of cancer patients.

## 1. Introduction

Cancer has been a significant public health concern worldwide for an extended period. Despite advances in medicine and the development of treatments for malignancies, the mortality rates of individuals diagnosed with cancer remain high, and the experience of cancer is still associated with discomfort, suffering, pain, and death.

According to the Joint Research Centre of the European Commission on the Burden of Cancer in European countries in 2020, the burden of cancer increased to 2.7 million new cases and 1.3 million deaths [1].

Numerous studies highlight the role of subjective well-being as extremely important for the sustainability of a healthy life and work [2]. Also, research from the post-COVID era additionally emphasizes the importance of well-being and mental health [3]. The subjective well-being of individuals affected by cancer is substantially compromised due to physical and psychological changes caused by surgical treatment, chemotherapy, radiotherapy, or hormone therapy [4,5]. Those diagnosed with cancer are continuously exposed to various stressors that undermine their psychological health and, consequently, their subjective well-being. These stressors are closely linked to the disease itself (treatment-related consequences, loss of physical functions, and body image issues), stressors arising from social relationships (financial problems due to the inability to continue working, feelings of loneliness, lack of social support, and problems in family relationships), and existential stressors (fear of dependence on others, fear of death, and a loss of a sense of purpose) [6]. Furthermore, research dealing with caregivers of people with chronic illnesses found that the diagnosis brings a significant change in the life of the whole family, which may lead to depressive symptoms [7,8].

Numerous studies indicate that received social support predicts higher levels of overall subjective well-being and that there is a significant association between life satisfaction and support received from significant individuals in an individual’s life [9,10]. Furthermore, according to the eudaimonic paradigm of well-being, the purpose in life is one of the six facets of psychological well-being [11,12,13]. Hence, purpose in life is an important factor in overall psychological well-being in both the general population and the population of individuals with a cancer diagnosis.

While we may have varying expectations, assuming that cancer patients exhibit compromised resilience due to prolonged exposure to stress or increased resilience through adaptation, existing research in this population reveals no significant difference in resilience levels compared to healthy individuals [14]. Moreover, studies examining the relationship between resilience and quality of life consistently show a positive association. For instance, in a study conducted on individuals suffering from various chronic illnesses, psychological resilience was found to act as a protective factor for subjective well-being [15]. Similar results were found in another study on women with breast cancer, where resilience emerged as a significant predictor of well-being [16]. In addition, cancer patients with high levels of resilience exhibited lower levels of anxiety and depression, better physical, emotional, and social functioning, and overall a higher quality of life than those with low levels of resilience [17].

Providing information to patients suffering from malignant diseases is one of the most important factors in supportive care for patients [18,19,20,21]. The goal of providing information is to prepare the patient for their treatment, encourage acceptance of the recommended treatment, increase their own abilities to cope with the cancer, and promote recovery [19]. The requirements for receiving information in cancer patients vary based on factors such as gender, age, cultural environment, level of education, type and degree of cancer spread, and coping styles [19]. The provision of information about the cancer itself and its treatment forms the foundation of supportive care for patients. Offering pertinent information to patients brings several advantages, including joint decision-making, increased satisfaction with care, enhanced patient control, reduced affective distress, improved communication with the patient’s family, and an overall better quality of life [18].

Patient-oriented communication is more effective when health professionals value the patient’s opinion on the received information. Patients with cancer do not always report that they have received enough information. In addition, clinicians’ efforts in providing information and patients’ expectations about receiving information may not always match, and patients’ expectations can change over time [18]. By providing adequate information to patients, it is possible to reduce the psychological burden and increase the quality of the patient’s life and their satisfaction with their care. Patients who are well informed about cancer, treatment, and subsequent care will finish treatment sooner and be less anxious later [21].

In previous research, it has been demonstrated that individuals diagnosed with cancer have a strong desire for information regarding the specific type of cancer, the side effects of chemotherapy, and the treatment prognosis [22]. They also express a need to know more about various aspects of care [23]. However, studies have shown that they receive a substantial amount of information from their specialized oncologists about the disease’s characteristics but receive less information about more sensitive topics such as prognosis and treatment outcomes [23]. Nonetheless, the statistics reveal that the majority of those affected (70.3%) are dissatisfied with the existing options for obtaining information about their illness [22]. However, the relationship between the level of information and personal well-being is still inadequately explored.

Numerous studies have investigated the predictive roles of optimism, internal health locus of control, self-confidence, purpose in life, and perceived social support in the subjective well-being of individuals undergoing chemotherapy [24,25,26,27]. Social support and purpose in life have also been identified as significant predictors of overall psychological well-being [28]. Reviewing previous research on dissatisfaction with information provided by medical professionals reveals that the level of information about the disease and related topics may play a crucial role in an individual’s well-being [22]. Research indicates that a higher level of information in cancer patients is linked to better adherence to medical instructions and treatment plans [29]. Based on this, it is evident that there are factors that can contribute to enhancing the subjective well-being of individuals diagnosed with cancer. The Biopsychosocial Model serves as a comprehensive lens, elucidating how the biological aspects of cancer interact with psychological processes, including the pursuit of purpose in life and adaptive coping strategies, while also recognizing the pivotal role of social support networks in fostering subjective well-being and resilience among cancer patients [30,31]. This study aims to explore the relationship between social support, purpose in life, resilience, and information levels with various components of subjective well-being in individuals diagnosed with cancer in Croatia. Previous research has indicated a significant association between resilience and life satisfaction, as well as between purpose in life and life satisfaction. However, the specific role of purpose in life as a potential mediator in the relationship between resilience and life satisfaction remains less explored. By employing mediation analysis, we aim to provide an understanding of how purpose in life may operate as a pathway through which resilient individuals experience enhanced life satisfaction [32,33,34,35]. In addition, the study will evaluate the potential to predict specific components of subjective well-being in individuals diagnosed with cancer based on these factors.

## 2. Materials and Methods

### 2.1. Participants

The study involved 338 individuals diagnosed with cancer, comprising 199 female participants and 139 male participants. The average age of the participants was 42.5 years (M = 42.5; SD = 12.45). Of the total number of participants, 62.1% were employed and 37.9% were not in active employment. Additionally, 74.9% of participants had a marital partner, while 24.9% were not in a marital relationship. Consequently, participants who were not in a marital relationship at the time of questionnaire completion did not respond to the subscales related to social support within the context of a marital partner.

**Figure 1:**
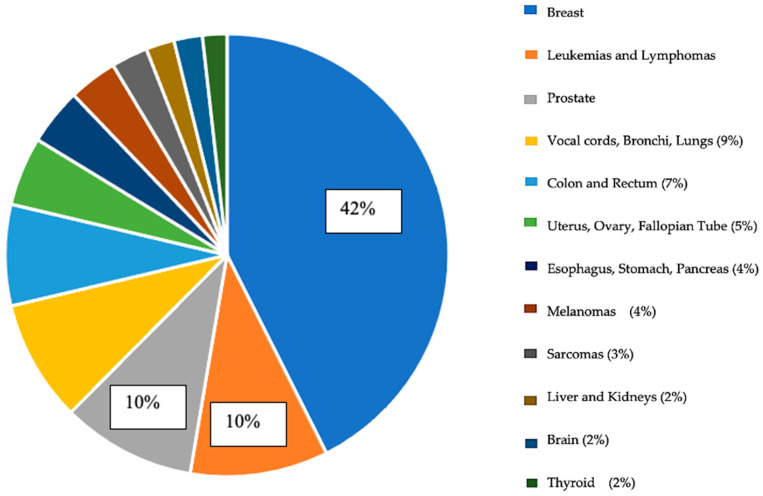
Graphic representation of the primary cancer sites among study participants (N = 338).

With regard to the primary site of cancer, most participants had diagnoses related to breast cancer, leukemia, lymphoma, and prostate cancer (Figure 1). The majority of participants had cancer at the second stage (33.4%), third stage (22.5%), and first stage of the disease (21.3%), with a smaller number of participants having cancer at the fourth stage of the disease (6.5%). Regarding their current health status, the majority of participants reported remission (49.7%), chronic conditions (21.9%), or an active treatment process (21.3%), while a smaller number of participants indicated recurrence (1.8%).

### 2.2. Instruments

#### 2.2.1. Sociodemographic Data and General Disease Information

Participants provided sociodemographic information about their age, gender, place of residence, marital status, and employment status. Additionally, general information about the disease was gathered, including the primary cancer site, cancer stage, and current disease status.

#### 2.2.2. Diener’s Scales of Subjective Well-Being [36]

To collect information about participants’ subjective well-being, a Croatian adaptation of Diener’s Scales of Subjective Well-Being was used, consisting of the Life Satisfaction Scale, the Positive and Negative Experience Scale, and the Prosperity Scale [37].

The Life Satisfaction Scale comprises five items measuring cognitive assessments of satisfaction with one’s own life. Participants were asked to rate their agreement with each statement on a scale from 1 (Strongly Disagree) to 7 (Strongly Agree). The total score is obtained by summing the responses to all five statements, with higher scores indicating greater life satisfaction.

The Positive and Negative Experience Scale consists of 12 items divided into two subscales. Six items measure positive feelings, and the remaining six items measure negative feelings. Participants rated their experiences over the past four weeks on a five-point scale (1—Very Rarely or Never, 5—Very Often or Always).

The Prosperity Scale comprises eight items describing aspects of human functioning. Participants were asked to express their agreement with each item on a seven-point scale (1—Strongly Disagree to 7—Strongly Agree). The total score is calculated by summing all ratings and can range from 8 to 56, with higher scores indicating greater perceived success in important areas of functioning. The study reported satisfactory reliability for the Life Satisfaction Scale (α = 0.86), Positive Experience Scale (α = 0.88), Negative Experience Scale (α = 0.84), and Prosperity Scale (α = 0.91).

#### 2.2.3. Workplace and Family Social Support Scale [38]

The Workplace and Family Social Support Scale consists of 36 items, organized into 4 sets of 9 items each, differing only by the source of support. It measures instrumental and emotional social support received from superiors at work, work colleagues, a marital partner, and other family members and close individuals. The analysis considered participants’ results on the Social Support Scale received from their partner and the Social Support Scale received from family members and other significant individuals. Each set of items contains five positively worded and four negatively worded items. Participants rated their agreement with each item on a seven-point scale (1—Strongly Disagree to 7—Strongly Agree). The total score on each subscale is calculated as the mean of the respective items. It is important to note that this scale assesses the intensity of perceived social support but not satisfaction with the received support. The study reported good reliability for the Social Support Scale received from a partner (α = 0.89) and the Social Support Scale received from family members and other significant individuals (α = 0.88).

#### 2.2.4. Purpose in Life Test [39]

The Purpose in Life Test (Croatian version) comprises 23 items assessing the emotional and cognitive aspects of a purpose in life [40]. Participants rated the extent to which the content of the statements applied to them on a five-point scale (1—Does Not Apply to Me at All to 5—Applies to Me Completely). The study reported a satisfactory level of reliability (α = 0.93).

#### 2.2.5. The Brief Resilience Scale [41]

The Brief Resilience Scale (Croatian version) consists of six items, with three positively worded items (e.g., “I usually bounce back quickly after hard times”) and three negatively worded items (e.g., “It is hard for me to snap back when something bad happens”). Participants rated their agreement with each item on a five-point scale (1—Strongly Disagree to 5—Strongly Agree) [42]. The study reported good reliability for the Resilience Scale (α = 0.85).

#### 2.2.6. EORTC QLQ-INFO25 [43]

The Information Scale includes four subscales that assess participants’ level of information about their illness, medical tests, treatment, and other medical services. Participants were asked to evaluate the amount of information they had received about their illness and treatment on a four-point scale (1—Not at all, 2—A little, 3—Quite a bit, 4—Very much). Additionally, this scale contains eight standalone items that examine areas not covered by the previous subscales, such as satisfaction with the amount of received information or the perceived usefulness of the information. The study reported a satisfactory overall reliability (α = 0.95).

### 2.3. Procedure

Convenience sampling was used to recruit participants. Permission for the research was obtained by the Department of Psychology, Faculty of Croatian Studies at the University of Zagreb (Zagreb, Croatia). Participants were recruited with the assistance of various associations for cancer patients and specialist oncologists who forwarded the invitation to participate in the research to their patients or association members. The associations and doctors were contacted through official email addresses. Data collection was conducted online using Google Forms survey forms from May 2022 to July 2022. All participants received the same instructions and provided informed consent to participate in the research.

The instructions emphasized the anonymity of participation in the research and the option to withdraw from completing the questionnaire at any time without consequences. The instructions also stated that the collected data would be analyzed at a group level and used exclusively for scientific purposes. Participants were asked to carefully read the instructions and were encouraged to answer the questions honestly in order to gather information about their experiences. As part of the instructions, participants were provided with the researchers’ email addresses for any questions or concerns.

## 3. Results

### Statistical Analysis

The statistical analysis of the results was conducted using the SPSS program version 22 (BM Corporation, Armonk, NY, USA). Descriptive analyses were performed on three aspects of subjective well-being (life satisfaction, positive and negative experiences, and prosperity), as well as a descriptive analysis of variables related to social support received from family members and other close individuals, social support received from a marital partner, purpose in life, resilience, and information.

Correlation analyses were utilized to examine the relationships between the components of subjective well-being and the psychosocial factors mentioned. Multiple regression analyses were carried out to determine the roles of individual psychosocial factors in explaining the variance in specific components of subjective well-being, separately for each criterion (life satisfaction, positive and negative experiences, and prosperity).

We also examined the potential mediating role of purpose in life in the relationship between resilience and life satisfaction. The analysis mentioned was conducted using PROCESS (version 4.0. for SPSS). A bootstrapping method was used to test the mediation effect, with 5000 bootstrap samples and the confidence interval (CI) set at 95%. The indirect effect of resilience on life satisfaction is considered significant if the range of CI (lower bound–upper bound) does not contain zero [44,45].

Basic descriptive data for the used variables were determined, and the normality of distributions was checked (Table 1). The Kolmogorov–Smirnov test showed a statistically significant deviation from normality for all variables except for the variable Informativeness. This result was expected, given the large number of different diagnoses within the same category.

Values on the subjective well-being scales indicated that the majority of participants were satisfied with their lives, had higher levels of prosperity, and more frequently experienced positive than negative experiences. According to the average values on the social support scale, participants perceived high levels of social support received from family and significant others, as well as social support received from their spouses. Higher values on the purpose in life scale indicated that the majority of participants had a sense of fulfillment and meaning. However, the results on the psychological resilience scale showed average values, suggesting that participants were not extremely resilient but also not completely sensitive to life stressors. Results on the information questionnaire indicated that participants were moderately informed about their illness, medical examinations, treatment, and other medical services.

**Figure 2:**
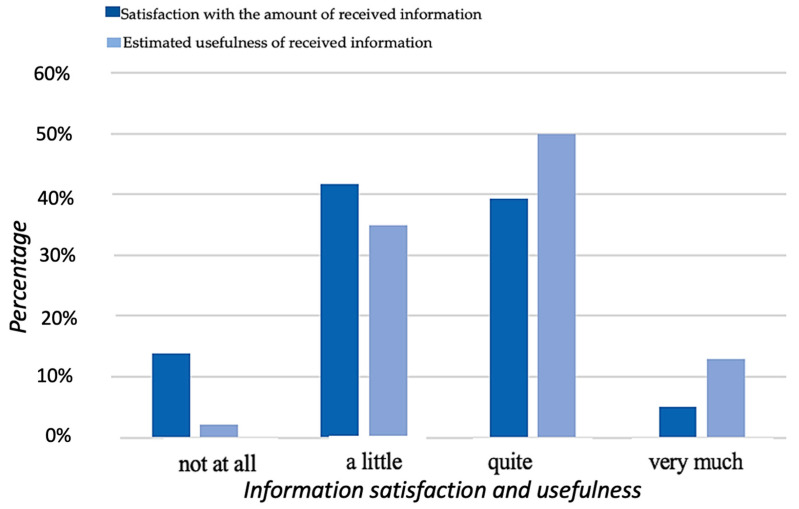
Graphical representation of response frequencies to items for Satisfaction with the Amount of Received Information and Estimated of the Usefulness of Received Information.

Most participants expressed that they were only slightly satisfied with the amount of information received from medical professionals, with very few being completely satisfied with the amount of information received (Figure 2). However, the majority found received information to be quite useful during and after treatment, whereas only a small number of participants found it useless.

A majority of participants (97.3%) indicated satisfaction with the information provided, while a minority (2.7%) expressed a desire for less information, specifically citing topics such as cancer patients’ survival rates and treatment prognosis, as highlighted by a subset of participants (n = 13).

**Figure 3:**
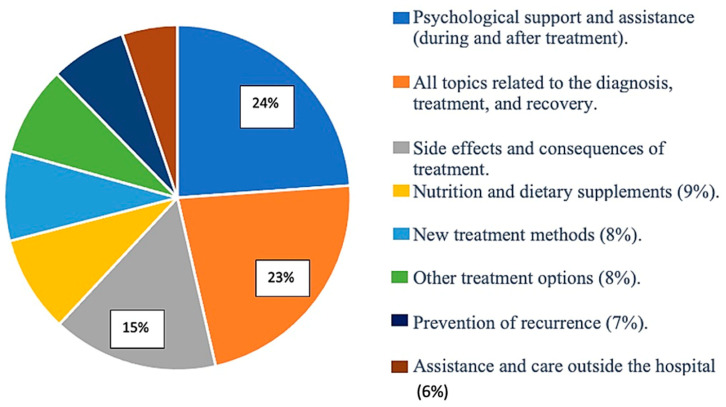
Graphical representation of thematic areas about which participants stated a desire for more information (n = 140).

Furthermore, 67.5% of the participants stated that they wanted to receive more information, while 32.5% stated that they did not want to receive more information about their illness, treatment, and other medical services. A total of 53% of participants had never received information about their illness in written form, and 86% of participants had never received information on a CD or videotape. Additionally, 140 participants (61.4%) from the group that expressed a desire for more information also mentioned specific topics they would like to receive more information about. Their responses were grouped into categories based on the topics they covered. Participants mentioned that they would like to receive more information about the following: (1) psychological support and assistance (during and after treatment), (2) all topics related to illness, treatment, and recovery, and (3) side effects and consequences of treatment (Figure 3).

Subsequently, the results of the correlation matrix and multiple regression analyses for the criteria of life satisfaction, positive and negative experiences, and prosperity are presented. These analyses were conducted on a subsample of participants who have a spouse (n = 253), allowing all predictors to be simultaneously included in the regression analysis. Individuals who do not have a spouse did not respond to items in the scale of social support received from a spouse. Hence, their responses cannot be included in the further analysis.

From Table 2, the interrelations among the three components of subjective well-being (life satisfaction, positive and negative experiences, and prosperity) can be seen. The variable of positive and negative experiences was negatively correlated with the variables of life satisfaction and prosperity. This means that higher levels of life satisfaction and prosperity were associated with more frequent positive experiences compared to negative experiences. Furthermore, life satisfaction showed significant low to moderate and positive associations with the variables of social support received from family and a partner, purpose in life, resilience, and information. Cancer patients who were more satisfied with their lives tended to have higher levels of purpose in life, perceived more social support from family and partners, had higher resilience to stress, and were more informed about their diagnosis, treatment, and medical services.

Participants who more frequently experience positive experiences, as opposed to negative ones, tend to have a stronger sense of purpose in life, perceive more social support, have higher resilience to stress, and are more informed about their medical condition.

Prosperity was significantly and positively correlated with other variables, indicating that individuals with higher levels of prosperity also tend to have a stronger sense of purpose in life, perceive more social support, have higher resilience, and tend to be better informed about their condition.

In order to examine the contribution of the observed variables (social support from family or marital partner, purpose in life, resilience, and informativeness) in explaining the three aspects of subjective well-being of individuals with cancer, multiple regression analyses were conducted (Table 3, Table 4 and Table 5). There were no deviations from the multicollinearity assumption between predictors of conducted regression analysis. The values of Variance Inflation Factor (VIF) and tolerance did not exceed critical values, i.e., the values of VIF ranged from 1.103 to 1.635, and of tolerance from 0.612 to 0.91.

The first regression analysis performed (Table 3) indicated the significance of the proposed model (F (5, 247) = 27.04; *p* < 0.001). All predictors included in the analysis explain 35.4% (*R*^2^*_adj_* = 0.341; *p* < 0.001) of the variance in life satisfaction. Purpose in life (β = 0.418, t = 6.39, *p* < 0.001) and social support from a marital partner (β = 0.235, t = 3.96, *p* < 0.001) were the only significant predictors, with purpose in life being the strongest one.

The multiple regression analysis with Positive and Negative Experiences as a criterion variable (Table 4) indicated the significance of the model (F (5, 247) = 62.68; *p* < 0.001). All predictors included in the analysis explain 55.9% (*R*^2^*_adj_* = 0.55, *p* < 0.001) of the variance of positive and negative experience, with purpose in life (β = −0.659, t = −12.2, *p* < 0.001) and resilience (β = −0.176, t = −3.61, *p* < 0.001) as the only variables that exhibit statistically significant predictive validity.

The multiple regression analysis conducted for Prosperity (Table 5) indicated the significance of the model, with 54.8% of the variance in the criterion variable explained by the observed set of predictors (F (5, 247) = 59.95; *p* < 0.001, *R*^2^*_adj_* = 0.539). The only significant predictor was purpose in life (β = 0.646, t = 11.81, *p* < 0.001).

The mediating role of purpose in life between resilience and life satisfaction was examined. Direct, indirect, and total effect results, as well as lower and upper confidence intervals for indirect effect, are presented in Table 6. The path coefficients from resilience to purpose in life, path a (B = 8.539, *p* < 0.001), and from purpose in life to life satisfaction, path b (B = 0.185, *p* < 0.001), were significant. Bootstrapped 95% confidence intervals indicated a significant indirect effect of resilience on life satisfaction through purpose in life (LLCI = 1.082, ULCI = 2.156). Hence, purpose in life mediated the relationship between resilience and life satisfaction. It should be noted that the total effect of resilience to life satisfaction was significant, path c (B = 2.144; *p* < 0.001); however, after including the mediated variable, the direct effect, path c’ (B = 0.566, *p* = 0.145), became insignificant.

## 4. Discussion

This study aimed to examine the relationships between various psychosocial factors and the subjective well-being of individuals diagnosed with cancer. The research was conducted with the objective of affirming established knowledge and advancing comprehension in this field, thereby offering practical implications for the improvement of subjective well-being among cancer patients. Our findings can provide new perspectives and guidelines for professionals working with cancer patients, thus directly assisting patients and clients in achieving a better quality of life.

### 4.1. The Prevalence of Subjective Well-Being and Psychosocial Factors

Consistent with the initial expectations, the research has shown that participants generally report satisfaction with their lives. The obtained results align with findings from other studies, which have shown that 50% of adult cancer patients express high life satisfaction, and that younger adult cancer patients also report above-average life satisfaction [46,47]. Similar results were found in samples of women with breast cancer and individuals with colorectal cancer [48,49]. Considering that cancer patients are continually exposed to various stressors that can potentially disrupt their overall functioning, it can be concluded that cancer patients successfully cope with these stressors, and they do not compromise their life satisfaction [6].

In relation to the average score on the prosperity scale, participants generally reported a high level of prosperity. The findings indicate that participants have fulfilled their universal psychological needs, such as the need for connection, competence, and self-acceptance [37]. High levels of prosperity suggest that participants are capable and competent in meeting personally important goals and needs, which is one of the indicators of high individual subjective well-being [50]. When these facts are considered in the context of cancer diagnosis, it can be inferred that participants mostly resist challenging and stressful circumstances, and these challenges do not prevent them from fulfilling important life goals and needs. Previous findings also demonstrate that the subjective well-being of cancer patients is not compromised when compared to a group of healthy individuals [51].

Participants predominantly experienced positive emotions in contrast to negative ones, which differs from the previous findings of the study that found a more frequent experience of negative emotions compared to positive ones among women with breast cancer [52]. The difference in results might be due to using Diener’s scale in this study, which measures general emotions. Other studies used scales capturing more complex emotions and experiences, like gratitude, hope, acceptance, empathy, and serenity, or contrasting emotions like shock, shame, helplessness, and “emotional block” [52,53].

Other studies focused solely on measuring negative experiences, i.e., the measurement of anxiety and depression levels in cancer patients, so we do not have information about positive experiences for comparison [54]. It should be noted that there were very few individuals in the sample who had stage IV cancer, where it is expected that the individual’s physical and mental condition is worse, and with it, there will be more frequent negative experiences.

Participants received a certain level of support derived from close relationships with others, but it seems that they believed they could receive even more. It is worth noting that the measurement pertained to the intensity of received support, leaving the question of participants’ satisfaction with it unanswered [38]. Received support was assessed in terms of concrete assistance (action; instrumental support) from close individuals, as well as emotional support in the form of understanding, acceptance, and comfort.

The participants’ age range was from 20 to 75 years, encompassing adult participants in various stages of life. They also differed in terms of marital status, employment status, primary site of cancer diagnosis, the stage of their disease at the time of study participation (stages I–IV), and the phase of treatment or recovery. Despite these differences among participants, the results indicated a high level of purpose in life, emphasizing its importance and serving as an incentive for deeper future research into personal meaning in individuals diagnosed with cancer.

Participants’ ability to successfully recover from stressful situations was at a moderate level. They were not exceptionally sensitive to stress and challenging circumstances, but they did require a certain amount of time for recovery. The obtained results were consistent with findings from other studies, showing that cancer patients do not differ from healthy individuals in terms of resilience, and that there is no difference in resilience based on the stage of the disease [14,55].

An analysis of individual items from the awareness questionnaire showed that participants’ levels of information were not high, and there is room for improvement in providing useful information to patients.

### 4.2. The Relationship between Subjective Well-Being and Psychosocial Factors

Significant moderate to high correlations were found between the constituent variables of subjective well-being (life satisfaction, positive and negative experiences, and prosperity) [37]. A positive direction of association was confirmed between the variables of life satisfaction and prosperity, while a negative direction of association was observed between the variable combining positive and negative experiences and the remaining variables [37]. Associations were also found between life satisfaction and various factors (social support, purpose in life, resilience, and information awareness). These findings are consistent with the results of other research, confirming a moderate to high positive association between a purpose in life, and social support with life satisfaction in a sample of cancer patients [10,56].

A negative association was found between the variable representing positive/negative experiences and psychosocial factors. Given that this variable combines the results of the Positive Experiences Scale and the Negative Experiences Scale, this result was expected. It indicates that a higher number of positive experiences (and, concurrently, fewer negative experiences) are associated with higher received support, a sense of purpose, levels of resilience, and information awareness. These findings are in line with the results in previous research [57].

Prosperity is most strongly associated with a purpose in life, but is also linked to other factors. These results align with the initial expectations, indicating that prosperity is associated with positive life experiences, emotions, and feelings of fulfillment and acceptance. The predictors used in this study do not explain a significant portion of the variance in life satisfaction. However, as expected, the significant predictors of life satisfaction were found to be purpose in life and social support received from one’s spouse. These results confirm findings from previous research, which emphasized the importance of a purpose in life and social support in the study of subjective well-being in cancer patients [56,58]. Received social support was shown to be a significant factor in predicting higher levels of overall subjective well-being [9].

Resilience predicted more frequent positive experiences such as happiness, joy, and comfort. This is consistent with previous research, where resilience was found to be a significant predictor of psychological well-being in a sample of women with breast cancer [16]. In addition to resilience, which emerged as a significant predictor, a high level of importance was attributed to purpose in life. It has been shown that individuals diagnosed with cancer who possess high levels of purpose in life tend to experience fewer negative and stressful incidents, emphasizing the importance of a purpose in life in predicting both positive and negative experiences [57]. Contrary to initial expectations, none of the forms of social support emerged as significant predictors in explaining the experiences of both positive and negative aspects. The reason for this might be that this study measured the intensity of social support received from family members and partners rather than the satisfaction with the support received [38]. Satisfaction with received support might play a more crucial role in experiencing positive events more frequently than merely the quantity of support received. This finding underscores the value of investigating satisfaction with support in individuals with cancer to gain a better understanding of the relationship between social support and the experiences of positive and negative aspects.

The level of informativeness did not prove to be a significant predictor of the experiences of positive and negative aspects. Once again, the reason for these results may be that this regression analysis examined the level of informativeness rather than the satisfaction with the level of information. For instance, a substantial amount of information received might help some individuals better understand their physical condition, thereby helping them to cope with their diagnosis and treatment. However, others might find an extensive amount of received information overwhelming [23].

In analyzing the results for the final component of subjective well-being, prosperity, it was possible to explain more than half of the variance in prosperity using the set of predictors. Individuals who have a high purpose in life are more likely to fulfill other psychological needs, such as competence, acceptance, and self-acceptance, thus experiencing full psychological prosperity. Nevertheless, the factors of resilience and informativeness did not appear to be significant predictors of prosperity in this study. This result does not align with other findings, indicating that resilience is a critical protective factor of subjective well-being [15]. The discrepancies might be attributed to methodological shortcomings and limitations in the study.

Our findings align with previous research suggesting that individuals with higher resilience tend to have a stronger sense of purpose, ultimately contributing to greater life satisfaction [32,33,34,35]. Purpose in life serves as a meaningful mediator, elucidating how resilience positively influences life satisfaction.

Interestingly, our study reveals a shift in in the relationship between resilience and life satisfaction after incorporating purpose in life as a mediator. This interplay emphasizes the importance of purpose in life in explaining how resilience influences life satisfaction. These findings offer additional insights compared to previous studies, highlighting the need for a comprehensive understanding of the complex dynamics between psychological resilience, purpose in life, and overall life satisfaction.

### 4.3. Limitations, Implications, and Recommendations for Future Research

The primary issue to address pertains to the cross-sectional design and the limitations of multiple regression analysis. Specifically, one must contemplate the potential bias in the results due to the inclusion of the informativeness factor, which exhibits very low correlations with the remaining variables (criteria and predictors). An improvement suggestion may involve the inclusion of the participant’s satisfaction variable with the quantity and quality of received information in the analysis. Future research could employ a longitudinal design in order to monitor potential changes of these aspects over time.

Given the limited external validity as a fundamental concern of multiple regression analysis, using a convenience sample can lead to the instability of regression coefficients and pose a threat to the validity of the obtained results. Another validity threat related to the participants in this study was the potential (un)conscious need for self-presentation and the display of socially desirable behaviors. Convenience sampling also presents a limitation since it could have led to the selection bias.

Despite the aforementioned limitations, this study holds certain theoretical and practical implications. Based on a literature review, this work can be considered the first in Croatia to consolidate various psychosocial factors of the subjective well-being of cancer patients. It can serve as an initial assessment of subjective well-being in this specific population and provide an overview of the important psychosocial factors of this construct. Given the comprehensiveness and elaboration of the subjective well-being construct into three components and the analysis of five different factors, it can undoubtedly serve as a foundation for future research in this field. The obtained results indicate a relatively high subjective well-being among cancer patients in Croatia, but there is always room for improvement, as it concerns a generally malleable construct among individuals in vulnerable life circumstances.

These theoretical insights can be utilized in the education of all healthcare staff who play a significant role in the treatment of cancer patients. They can also benefit family members as well as cancer patients by providing education and developing various skills to achieve the highest possible subjective well-being, to improve overall quality of life, and to extend the lifespan for cancer patients.

## 5. Conclusions

This study revealed high levels of life satisfaction and prosperity, coupled with a more frequent occurrence of positive experiences compared to negative ones. The findings indicate a noteworthy level of subjective well-being among cancer patients in Croatia. The research also identified a moderate to high level of social support received from family members and other significant individuals, along with a moderate to high level of support from spouses.

High levels of purpose in life were observed, as well as a moderate level of psychological resilience and awareness regarding the disease, screenings, treatment, and other forms of assistance. Furthermore, a strong interrelationship was found among the components of the subjective well-being construct, while low to moderate correlations were identified among different psychosocial factors. Positive associations were observed between life satisfaction and various factors (meaning in life, social support, resilience, and awareness), as well as between prosperity and the mentioned psychosocial factors.

Purpose in life and social support from spouses emerged as the strongest predictors of life satisfaction. Purpose in life and resilience were identified as the primary factors explaining both positive and negative experiences, while purpose in life was the most influential predictor of prosperity among cancer patients. In conclusion, purpose in life stands out as an exceptionally significant predictor of subjective well-being among cancer patients, followed by social support from spouses and the psychological resilience of participants. Notably, social support from family and close individuals, as well as the participants’ level of awareness, did not prove to be significant predictors of subjective well-being in this study. Purpose in life plays a mediating role between resilience and life satisfaction.

These findings not only inform the education of healthcare professionals and family members caring for cancer patients, but also directly benefit the patients by raising awareness, fostering understanding, and providing guidance for psychotherapeutic interventions, ultimately aiming to maximize subjective well-being, expedite recovery, and enhance overall quality of life.

## Figures and Tables

**Table 1:** Basic descriptive data, results of normal distribution, and distribution parameters for psychosocial variables and subjective well-being variables.

Variable	N	M	SD	Min	Max	KS	Skewness	Kurtosis
Life Satisfaction	338	24.65	5.94	5.00	35.00	0.093 **	−0.78	0.50
PN Experiences	338	−7.11	7.98	−24.00	22.00	0.069 **	0.47	0.34
Prosperity	338	44.97	7.63	22.00	56.00	0.081 **	−0.63	−0.15
Social Support (F)	338	5.35	1.12	1.78	7.00	0.100 **	−0.43	−0.60
Social Support (P)	253	5.64	1.23	1.44	7.00	0.133 **	−0.95	0.40
Purpose in Life	338	90.39	15.11	32.00	115.00	0.125 **	−0.77	0.16
Resilience	338	3.19	0.82	1.00	5.00	0.077 **	−0.21	0.22
Informativeness	338	46.41	12.93	6.48	83.33	0.050	0.33	0.27

Note: ** *p* < 0.01, PN—Positive and Negative, F—Family, P—Partner, KS—Kolmogorov–Smirnov test.

**Table 2:** Correlations of social support factors (family and partner), purpose in life, resilience, and information with components of subjective well-being.

	1.	2.	3.	4.	5.	6.	7.	8.
1. Life satisfaction	-	−0.527 **	0.563 **	0.339 **	0.380 **	0.551 **	0.331 **	0.171 **
2. PN experiences		-	−0.712 **	−0.314 **	−0.261 **	−0.731 **	−0.470 **	−0.202 **
3. Prosperity			-	0.441 **	0.298 **	0.732 **	0.384 **	0.183 **
4. Social support (F)				-	0.486 **	0.464 **	0.226 **	0.115
5. Social support (P)					-	0.316 **	0.107	0.130 *
6. Purpose in life						-	0.478 **	0.262 **
7. Resilience							-	0.246 **
8. Informativeness								-

Note: * *p* < 0.05, ** *p* < 0.01, PN—Positive and Negative, F—Family, P—Partner.

**Table 3:** Regression analysis results for the criterion of life satisfaction.

	Unstandardized Coefficients		Standardized Coefficients		
Model	*B*	Standard Error	β	*T*	*p*
Constant	3.483	2.012		1.731	0.085
Social Support (F)	0.052	0.319	0.01	0.164	0.87
Social Support (P)	1.022	0.258	0.235	3.962	<0.001
Purpose in life	0.152	0.024	0.418	6.391	<0.001
Resilience	0.642	0.382	0.099	1.678	0.095
Informativeness	0.001	0.022	0.001	0.027	0.979
*R*	*R* ^2^	*Adjusted R* ^2^	Standard Error	*F*	*p*
0.595	0.354	0.341	4.338	27.042	<0.001

Note: F—Family, P—Partner.

**Table 4:** Regression analysis results for the criterion of positive and negative experiences.

	Unstandardized Coefficients		Standardized Coefficients		
Model	*B*	Standard Error	β	*T*	*p*
Constant	30.137	2.492		12.092	<0.001
Social Support (F)	0.523	0.395	0.069	1.325	0.186
Social Support (P)	−0.47	0.319	−0.072	−1.471	0.143
Purpose in life	−0.361	0.03	−0.659	−12.202	<0.001
Resilience	−1.709	0.474	−0.176	−3.607	<0.001
Informativeness	0.011	0.027	0.017	0.392	0.695
*R*	*R* ^2^	*Adjusted* *R*^2^	Standard Error	*F*	*p*
0.748	0.559	0.55	5.373	62.684	<0.001

Note: F—Family, P—Partner.

**Table 5:** Regression analysis results for the criterion of prosperity.

	Unstandardized Coefficients		Standardized Coefficients		
Model	*B*	Standard Error	β	*T*	*p*
Constant	9.835	2.305		4.266	<0.001
Social Support (F)	0.767	0.365	0.111	2.101	0.037
Social Support (P)	0.23	0.296	0.039	0.78	0.436
Purpose in life	0.323	0.365	0.646	11.807	<0.001
Resilience	0.46	0.027	0.052	1.049	0.295
Informativeness	−0.01	0.438	−0.018	−0.408	0.684
*R*	*R* ^2^	*Adjusted* *R*^2^	Standard Error	*F*	*p*
0.74	0.548	0.539	4.9698	59.951	<0.001

Note: F—Family, P—Partner.

**Table 6:** Mediation analysis results of resilience on life satisfaction through purpose in life.

Predictor (X)		M (Purpose in Life)			Y (Life Satisfaction)		Indirect Effect 95%CI	
		B	SE		B	SE	Lower Bound	Upper Bound
Resilience	a ***	8.539	0.986	c	0.566	0.387		
				c ***	2.144	0.385		
Purpose in life				b ***	0.185	0.022	1.082	2.156
		*R*^2^ = 0.229			*R*^2^ = 0.31			
		F(1.252) = 74.95, *p* < 0.001			F(2.251) = 56.32, *p* < 0.001			

Note: SE—standard error; B—unstandardized coefficients, CI—confidence interval, *** *p* < 0.001.

## Data Availability

The data presented in this study are available on request from the corresponding author.
